# Novel Simulation With 3D-Printed Spine for Teaching Durotomy Repair: A Technique Guide and Validation Study

**DOI:** 10.1097/SIH.0000000000000889

**Published:** 2026-01-15

**Authors:** Lainey G. Bukowiec, Aaron Damon, Julia Todderud, Seung J. Lee, Paul Huddleston

**Affiliations:** Mayo Clinic Department of Orthopedic Surgery, Rochester, NY (L.G.B., A.D., J.T., P.H.); Mayo Clinic Department of Neurological Surgery, Jacksonville, FL (S.J.L.)

**Keywords:** Durotomy, surgical simulation, orthopedic surgery, neurosurgery, spine surgery, spine

## Abstract

**Introduction:**

Durotomies can lead to cerebrospinal fluid leakage, resulting in various complications. Repairing a durotomy is an essential skill for orthopedic and neurosurgical trainees, but the learning process can be challenging. Therefore, there is a need for effective simulators that allow surgical trainees to practice the technique in a controlled environment without endangering patient safety.

**Methods:**

This study describes the development of a novel 3-dimensional (3D) printed simulation model designed for teaching the repair of intraoperative durotomies to surgical trainees. Junior and senior residents, fellows, and attending spine surgeons used the simulation. Inter- and intragroup performance was examined.

**Results:**

A novel durotomy repair simulation model with lumbar and thoracic windows was developed using 3D-printed and repurposed components to create a lifelike representation of an in vivo intraoperative scenario. Senior residents, fellows, and attendings outperformed junior residents at the initial attempt. All groups improved with repeated exposure to the simulator.

**Conclusions:**

The model offers surgical trainees a controlled environment to practice technical skills without increasing risk for patients or prolonging surgical cases. The model demonstrated its validity by showing that more senior participants outperformed junior residents during their initial attempts. Performance improvement across all groups with repeated exposure indicates that the model not only tests a relevant and realistic skill set, but also facilitates skill development over time. Given the challenges associated with intraoperative training as well as the complications associated with durotomies, the proposed simulator has the potential to benefit surgical trainees and patients.

Surgical simulation has evolved over the years and has become an important part of surgical education. Traditionally, students used box trainers to learn basic surgical skills, but the development and evolution of 3D printing have allowed more anatomically accurate models for learning.^1^ Surgical simulation training has been shown to enhance patient outcomes.^2^,^3^ Simulation allows residents to progress rapidly through the learning curve of skill acquisition and acquire a greater understanding of technique and decision-making.^4^ Surgical simulation training, including benchtop trainers, animal models, simulated operating rooms, and virtual/augmented reality, allows learners to refine skills by practicing advanced patient scenarios.^5^

Surgical simulation plays a crucial role in residency and fellowship training. Metrics used to evaluate the quality of resident and fellow education, such as case volume and level of participation, can vary significantly among training programs. Intraoperative training of residents and fellows poses challenges, including potential risks to patients.^6^ The presence of a trainee learning a skill during surgery can prolong the duration of the procedure and increase the risk of complications. Surgical simulation offers a solution by providing a controlled setting outside of the operating room to prepare residents for real-life scenarios, mitigating risks associated with intraoperative training.^7^

Durotomies, the unintended tearing of the protective covering of the spinal cord, can occur during spinal surgery. Durotomies can lead to cerebrospinal fluid leakage, resulting in various complications.^8^–^11^ Repairing a durotomy is an essential skill for orthopedic and neurosurgical residents, but the learning process can be challenging. Therefore, there is a need for effective simulators that allow surgical trainees to practice the technique in a controlled environment without endangering patient safety. A novel durotomy repair simulation model with lumbar and thoracic windows has been developed, incorporating a 3D printing technique to create a lifelike representation of an in vivo intraoperative scenario. This 3D printing technique would allow for custom, affordable, and realistic representations of models from patient imaging that are better able to accommodate variations in anatomy, patient, and surgical conditions.

## MATERIALS AND METHODS

### Imaging Acquisition

Following Institutional Review Board approval, a standard preoperative T1 magnetic resonance imaging with 1- to 2-mm cuts was employed to create a realistic spine model. The scan was anonymized to protect patient data. If available, an established institutional pathway for acquiring spinal imaging may be used. The scan was uploaded into open-access Digital Information and Communications in Medicine (DICOM) viewing software, 3D Slicer (3Dslicer Version 4.10.2 Windows, available for download at https://www.slicer.org).

### Segmentation of Vertebrae

An appropriate plane of view identifying the maximal diameter of each vertebra was selected using 3D Slicer software. The Segment Editor tool was used to render out the volume of individual vertebrae on multiple image slices. The Paint Tool was used to highlight the adjacent vertebra on at least 3 continuous cuts. 3D Slicer software could then generate an automated segmentation based on previously highlighted structures, converting it into a 3D image. Images were carefully inspected to ensure adequate capture of each vertebral level. The Erase Tool was used to modify the appearance if unwanted tissue was present. When the final image was of satisfactory quality, the image was saved and exported as a Standard Tessellation Language (STL) file.

### File Editing

A computer-assisted design tool, Meshmixer (Autodesk, Inc, version 3.5.0), was employed for further file editing and model creation. STL files were uploaded into Meshmixer. The Analysis tab allowed for automated inspection of the vertebral segments to ensure completeness for printing. The files were then uploaded into a slicing software appropriate for the type of desktop 3D printer used (UltiMaker Cura Version 5.3.0, Product ID 218251–218256). For this model, an Ultimaker S5 dual-extrusion printer was used. Other options include the Ultimaker 3 and the MakerBot Replicator series, or single-extrusion printers as available. The given process was repeated for each individual vertebral segment of the spine. Spine segments were then printed, postprocessed, and assembled postproduction to demonstrate a full spine model.

### Model Creation and Assembly

**FIGURE 1:**
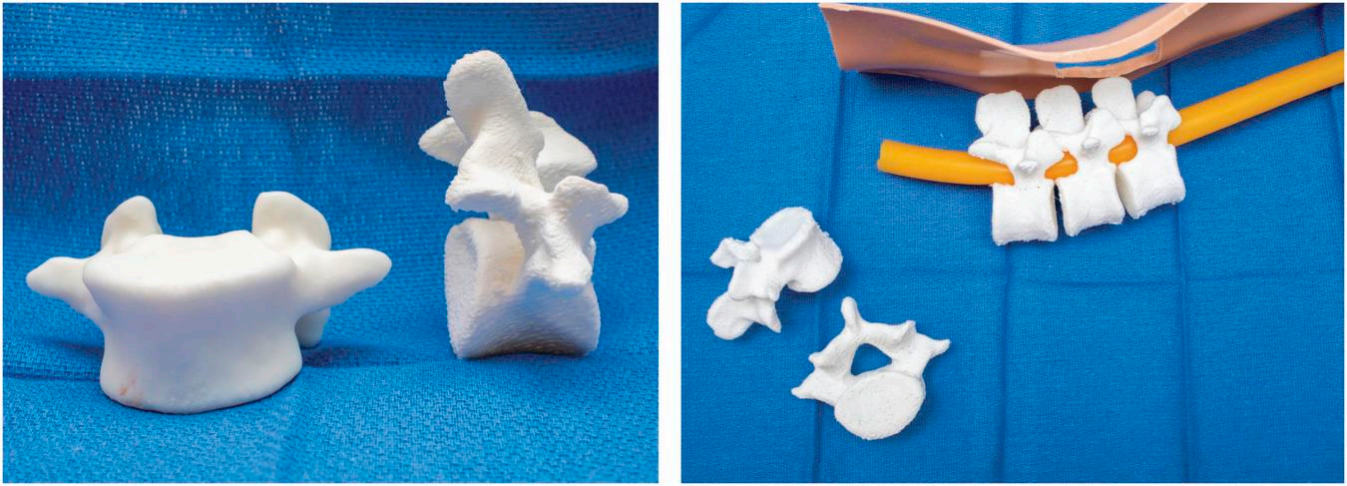
Assembly of vertebrae along a latex surgical tube, with a cut segment of simulated skin including a lumbar window.

Individual vertebrae were 3D printed using white acrylonitrile butadiene styrene (ABS) filament (MatterHackers, Lake Forest, CA) and assembled along a 1/2″ Inside Diameter (ID) × 1/16″ wall thickness (W) × 5/8″ outside diameter (OD) latex surgical tube to mimic the spine's structure (Fig. 1).

**FIGURE 2:**
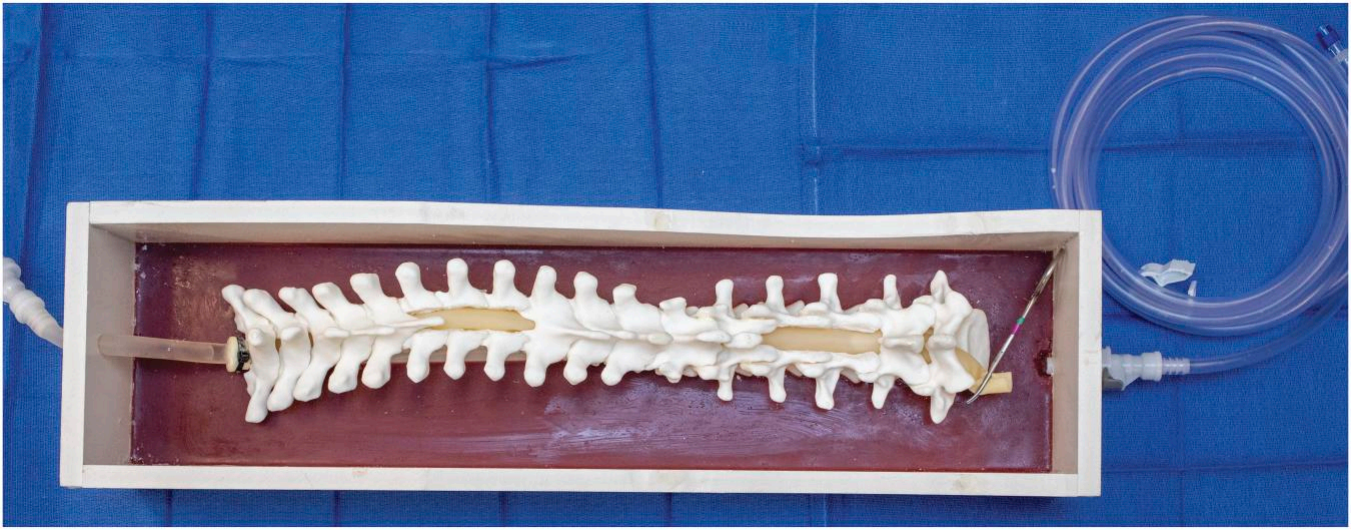
Spine model embedded in latex with a Penrose drain in place.

A housing box was custom-made using a high-density polyethylene board (48″ × 24″ × 1/2″ inches; King Plastic Corporation; North Port, Florida, US) to fit the final dimensions of the thoracolumbar spine model. Ecoflex 00-20 silicone (Smooth-On; Macungie, Pennsylvania, US) was colored with FuseFX silicone dyes (S-301-D and BC-03; Ottawa, Ontario, Canada) to imitate the color of muscle (Fig. 2). The assembled 3D models were then positioned into the high-density polyethylene housing unit. The housing box was filled up to 50% with dyed silicone and was cured for 12 hours. After curing, the latex surgical tubing was removed.

**FIGURE 3:**
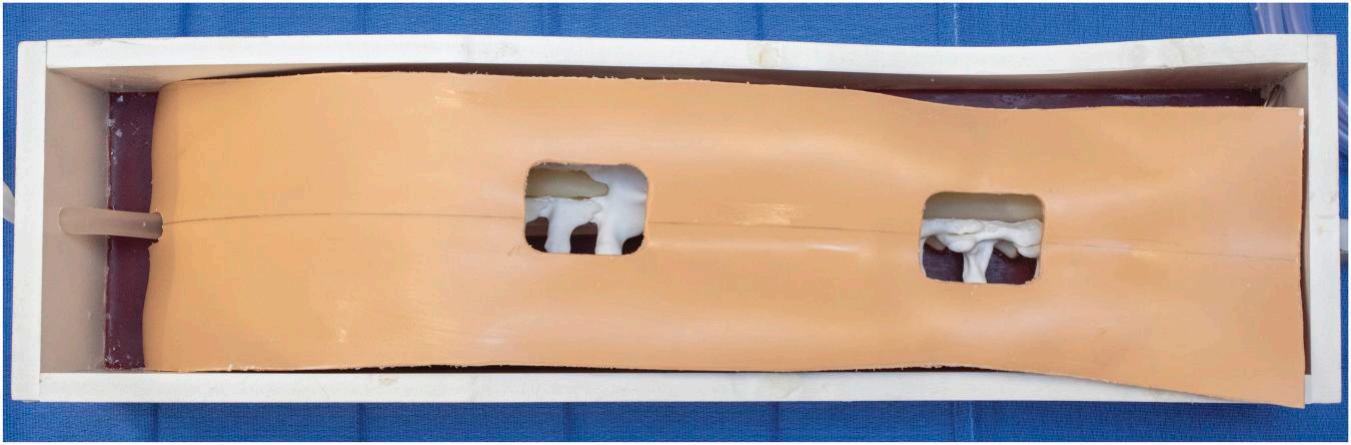
Spine model with overlying skin and surgical thoracic and lumbar windows.

Skin from an ABS polymer male 3/4 mannequin torso was cut to overlay the housing box. Small surgical windows were made in the skin to represent operative windows and add depth to the simulation (Fig. 3). While a polymer mannequin was used to simulate skin in the current model, any plastic material may be used to overlay the housing box, provided that an appropriately sized operative window is created.

**FIGURE 4:**
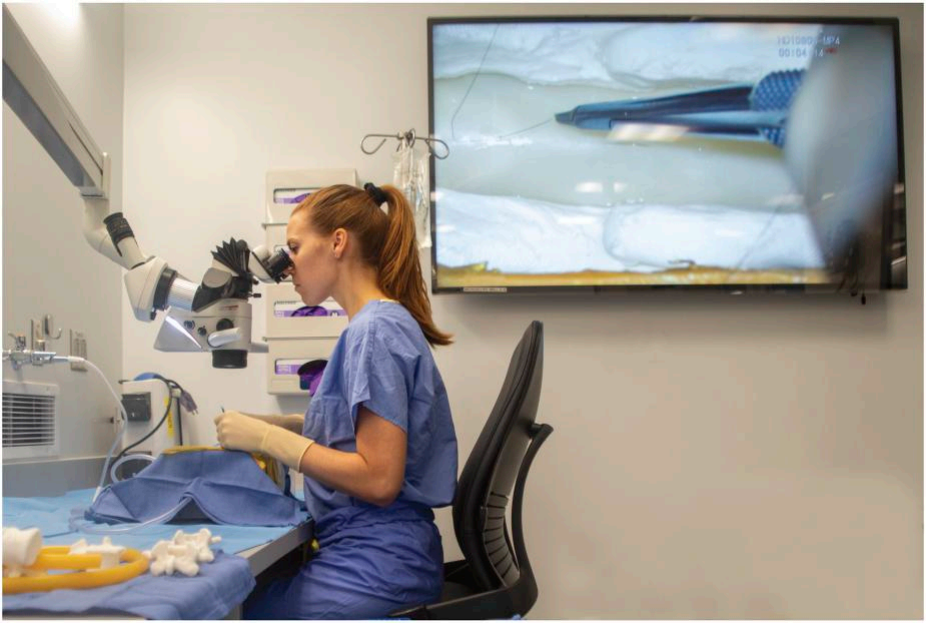
Surgical trainee practicing simulated durotomy repair with the screen displaying the view from a microscope.

**FIGURE 5:**
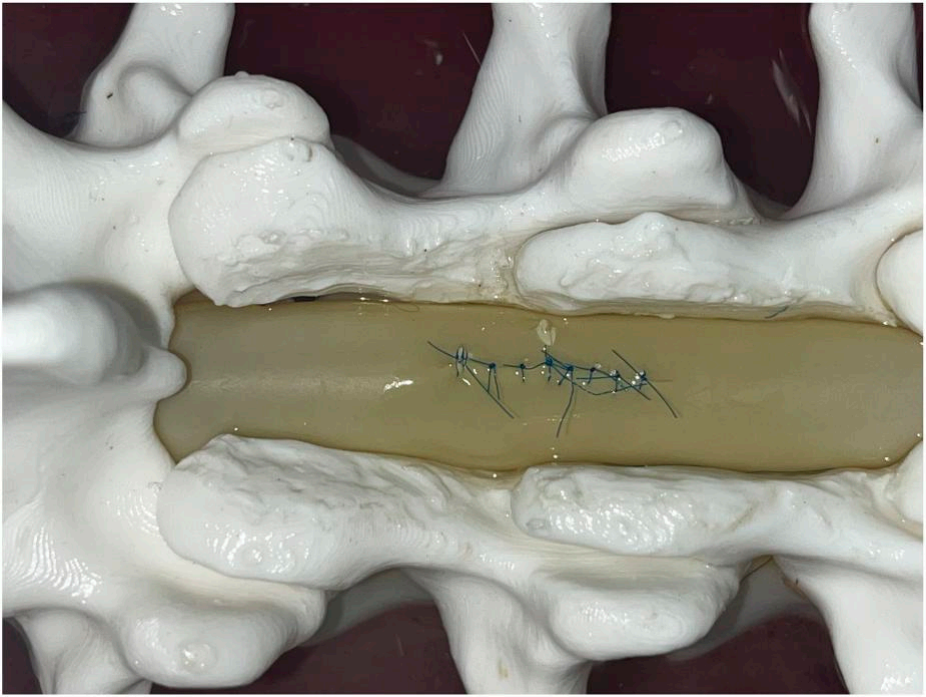
View through a microscope of attempted durotomy repair with leak present.

**FIGURE 6:**
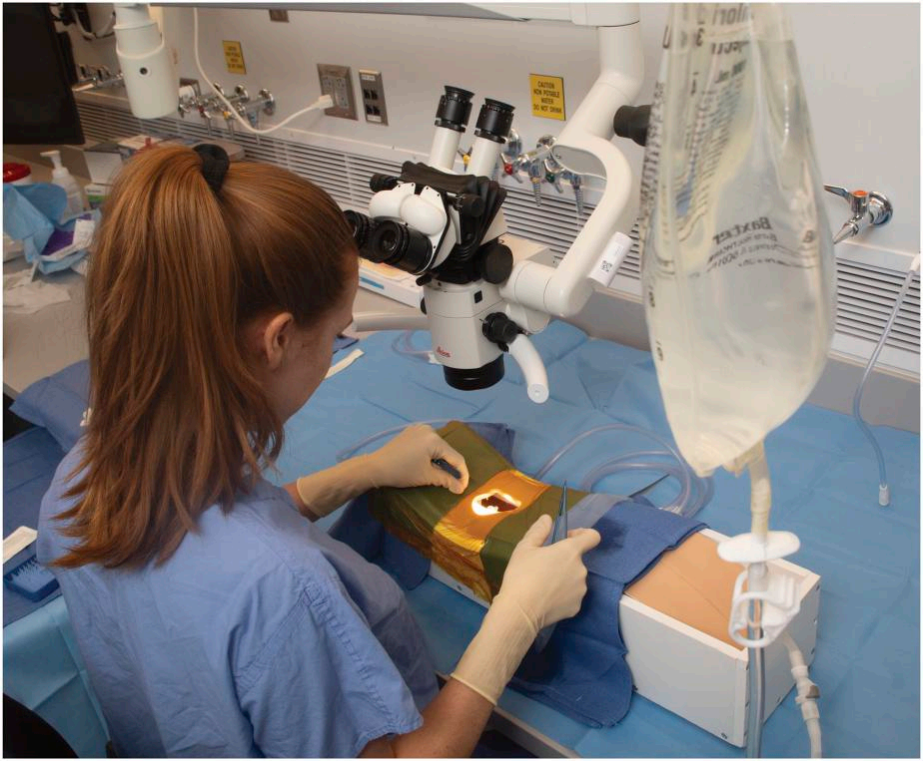
Simulated surgical field.

Using a high-speed drill, a laminectomy was performed on the model at the L2–L3 and T6–T7–T8 vertebrae to create laminar windows. To represent the spinal dura, a 5/8″ × 18″ Penrose drain was inserted into the spinal canal (Graham-Field, Atlanta, GA; Figs. 4 and 5). The Penrose drain can be tied or clamped at the inferior end. The drain at the superior end was affixed to an Iron star quick disconnect hose barb (Product ID 682604757025; Ironstar XDTEC, Shenzhen City, Longgang District, Guangdong Province, China) with a standard zip tie. A 3000-mL saline bag suspended on an IV pole was connected to the superior end of the Penrose drain (shown in Fig. 6). The rate of flow can be regulated with the IV roller clamp. These components serve as simulated cerebrospinal fluid with user-controlled intradural pressure based on flow rate of the saline. A small hole can be drilled in the inferior board of the housing box, in which excess saline can be washed out by attaching a suction device or by gravity.

### Clinical Validation

Residents of all levels, fellows, and board-certified attending spine surgeons were recruited via email to participate in the study. Participation was voluntary and without compensation. A prerecorded oral consent script was played for participants before commencing the study. Participants were timed with the same manual stopwatch while performing durotomy repairs using the durotomy simulator. All participants were instructed to use only simple interrupted sutures, and the same suture material and micro-instruments were provided to ensure consistency. Timing began when the participant picked up the suture and ended when they indicated completion of the repair. Residents and fellows completed 5 attempts, whereas attendings performed 3. Attendings were asked to complete fewer attempts because of limited availability and clinical responsibilities. In addition, requiring more repetitions from trainees allowed for a more sensitive assessment of skill acquisition and better differentiation between experience levels. Repairs were evaluated for success by a third-party observer who visually inspected whether a leak was present when the saline flow rate was increased to maximum speed after completion of the repair. A leak apparent to the naked eye indicated an unsuccessful repair. First and last attempt times of postgraduate year (PGY)-1, PGY-2, and fellows/attendings were compared using paired *t* tests. Because of the small sample sizes within the attending and fellow groups, these 2 categories were combined for the in-group analysis to increase statistical power and improve the reliability of comparisons. Senior residents were not examined for an in-group comparison as there were only 2 participants. The rates of successful repairs per attempt within each of the varying experience levels were also compared.

## RESULTS

A novel durotomy repair simulation model with lumbar and thoracic windows has been developed using a 3D printing technique to create a lifelike representation of an in vivo intraoperative scenario. The vertebrae STL files for 3D printing are available for free download on thingiverse.com under the heading “C2-L5 Anatomically Accurate Spine Model.”^12^ The simulator was formally tested by surgical residents, fellows, and board-certified spine surgeons. Demographic participant data can be found in Table 1.

**TABLE 1:** Demographic Information on Study Participants Including Sex, Age, Handedness, Exposure to the Instruments, and Interest in Microsurgery

Sex	Age	Year of Training	Handedness	Exposure to the Instruments?	Interest in MIS/Microsurgery?
M	30	PGY-1	R	N	Y
F	27	PGY-1	R	N	Y
F	26	PGY-1	R	N	N
M	27	PGY-1	R	Y	N
M	27	PGY-1	R	N	Y
M	30	PGY-1	R	Y	N
F	28	PGY-2	R	Y	Y
F	30	PGY-2	R	Y	Y
M	31	PGY-2	R	N	Y
F	28	PGY-2	R	Y	Y
M	30	PGY-2	R	Y	N
F	28	PGY-2	R	Y	Y
M	33	PGY-2	R	Y	N
M	29	PGY-4	R	Y	N
M	31	PGY-5	R	Y	Y
M	31	PGY-6	R	Y	Y
M	30	PGY-6	R	Y	Y
M	54	Attending	R	Y	Y
M	38	Attending	R	Y	Y
M	46	Attending	R	Y	Y
M	50	Attending	L	Y	Y

Participants practiced durotomy repair techniques and microsurgical skills as shown in Figures 4 and 6. The amount of time per attempt for each participant is included in Table 2.

**TABLE 2:** Time Per Attempt for Each Participant

Participant	Attempt Time (min)				
	1	2	3	4	5
PGY-1	28.48*	25.93*	25.48	21.45	23.32
PGY-1	38.15*	19.12	16.17*	24.08*	18.83
PGY-1	33.80*	23.90	23.82*	24.68	22.15*
PGY-1	26.57*	25.60*	20.73	20.02*	19.47*
PGY-1	20.38*	17.87*	17.42	16.00	13.33*
PGY-1	27.55*	33.25*	34.22*	23.30*	27.77
PGY-2	25.28	22.18	16.68	16.17*	9.75*
PGY-2	18.42*	17.30*	22.50	11.33*	13.17
PGY-2	9.28*	5.22*	12.05	11.25	8.93
PGY-2	30.13	28.15*	29.92*	31.02	25.53
PGY-2	23.50*	14.18	15.97*	16.82*	23.48*
PGY-2	21.55*	20.52*	13.25	12.40*	13.02
PGY-2	25.17*	20.68	23.42*	19.42	15.07*
PGY-4	16.27*	19.17	16.42	17.93*	17.45
PGY-5	13.82*	14.05*	13.42*	15.03	13.73*
Fellow	13.10	10.57	7.17*	13.53	12.38*
Fellow	12.88*	9.62	13.63*	8.55*	9.33
Attending	15.08	11.28	12.90		
Attending	13.23*	13.60*	8.38		
Attending	24.00*	22.85*	17.37		
Attending	16.72	17.82	14.92*		

*Unsuccessful repair as indicated by leak in simulated dura.

**FIGURE 7:**
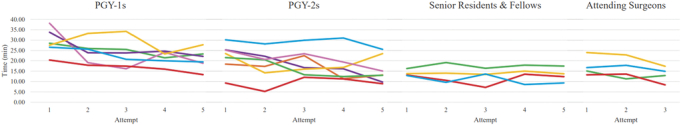
Time per attempt within each training level.

The in-group comparison of first versus last attempt times revealed significant improvements in performance across all examined groups (Fig. 7). For first-year residents, the average time decreased by 8.34 minutes from the first to the last attempt (SD = 6.60, *P* = 0.027). Second-year residents showed a similar improvement, with an average reduction of 6.34 minutes (SD = 5.53, *P* = 0.023). Fellows and attendings combined demonstrated a smaller but still significant improvement of 3.29 minutes (SD = 2.18, *P* = 0.014).

**FIGURE 8:**
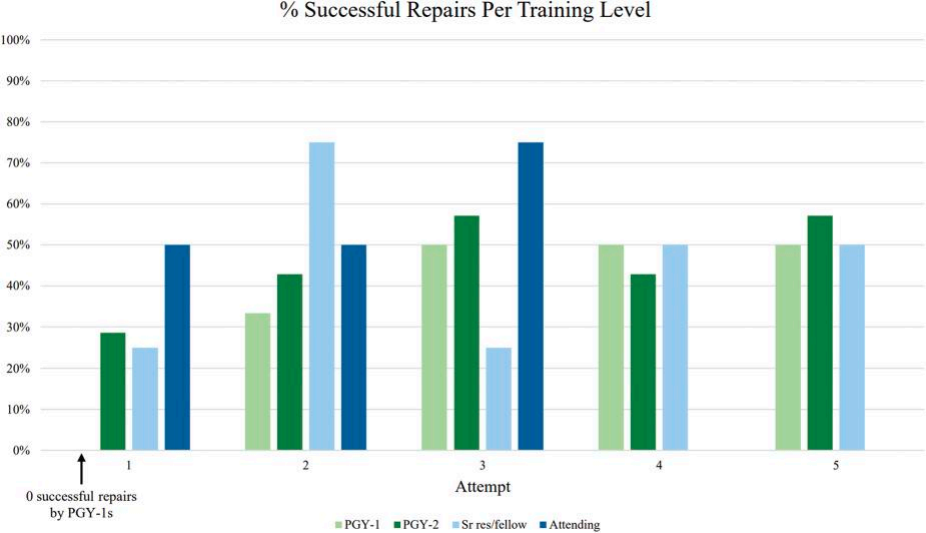
Successful repairs per training level.

Figure 8 displays percentage of successful repairs on each attempt. Notably, attending surgeons only completed 3 attempts, whereas all other groups completed 5 attempts. On the first attempt, success rate of durotomy repair as determined by absence of leak at attempt completion was 0% for PGY-1s, 29% for PGY-2s, 25% for senior residents and fellows, and 50% for attending surgeons. By the last attempt, the success rate was 50% for PGY-1s, 57% for PGY-2s, 50% for senior residents and fellows, and 75% for attending surgeons.

**TABLE 3:** Cost of Materials Required to Run the Simulation

Material	Cost (USD)
3D printer (one-time rental)^a^	$0.00
IV pole^a^	$0.00
Empty saline bag^a^	$0.00
Microsurgical tools^a^	$0.00
Mannequin torso (discarded CPR trainer) or any plastic material cut with an operative window^a^	$0.00
220-g ABS filament	$9.67
Dyed silicone (Ecoflex)	$45.66
Silicone tubing ½″	$1.49
Housing box (PVC board)	$36.22
Latex Penrose drain 18″ × 5/8″^b^	$1.39
Suture 6-0 polypropylene^b^	$8.33
Total build cost	$93.04
Total disposable usage cost	$9.72

^a^Items presumed readily available within a hospital setting.

^b^Items that need to be restocked after each use.

### Feedback From a Surgical Trainee

“As a junior resident with minimal experience in microsurgery and no prior exposure to repairing durotomies myself, the durotomy repair simulation proved to be an invaluable asset in my training. Through multiple sessions with the simulator, I experienced significant growth in both my technical skills and confidence levels. The fidelity of the simulation is remarkably high. It provides an immersive and realistic environment that closely mirrors what I have seen in the operating room. The ability to control intradural pressure and tailor the difficulty level by creating dural tears in different locations throughout the simulated dura has been instrumental in refining my microsurgical techniques. I firmly believe that the incorporation of this simulation model into medical training programs will not only enhance the skill acquisition process but also lead to improved patient outcomes by ensuring that trainees are well-prepared to manage challenging surgical scenarios.”

### Feedback From a Board-Certified Spine Surgeon

“As someone with many years of experience performing spine surgeries and educating future spine surgeons, I can confidently say this simulation tool is a game changer in surgical education. This model offers a simulation sanctuary where mistakes lead to learning rather than litigation or, more importantly, risks to patients. The level of realism is exceptional; it closely mimics the tactile sensations of working with delicate spine tissues. I am also highly encouraged by the improvement in learners' performance, both in terms of their surgical times and their overall facility with the technique. The real-time feedback provided by the device allows learners to identify and correct their mistakes, accelerating their mastery of the procedure. It's been truly rewarding to witness how quickly they develop competence in what is typically a very challenging skill.”

### Cost Considerations

The use of readily available materials and reusable 3D-printed materials suggests a relatively cost-effective solution compared with other simulation methods, such as virtual reality or cadaveric models. Model construction requires a 3D printer, ABS filament, latex surgical tubing, dyed silicone, a housing unit, a mannequin torso, Penrose drains, suture material, microsurgical tools, an IV pole, and an empty saline bag, which are generally less expensive than specialized simulation equipment (Table 3). The simulator is reusable, requiring only inexpensive materials such as suture and a Penrose drain to reset it for additional practice sessions.

## DISCUSSION

This study presents a novel 3D-printed simulation model for teaching durotomy repair, achieving the design objectives of creating a lifelike intraoperative scenario for surgical training. The implementation details, formal testing, and feedback from participants demonstrate the simulator's potential to enhance surgical education. Use of 3D printing allows for variations in spine models, at a reduced cost relative to marketed simulators. The Penrose drain simulates the dura with readily available and cost-efficient materials to decrease barriers for training while maintaining simulator fidelity.

Based on the validation study, the 3D-printed simulation appears to be a valid task for improving the technical ability of surgical trainees. The fidelity of the simulator to a real-life operative durotomy is suggested by the superior performance of more experienced participants on first attempt. For example, on first attempt, 0% of PGY-1 had a successful repair, whereas 50% of attendings successfully repaired the leak. On last attempt, the groups had more similar rates of successful repairs, closing the gap among experience levels. The validity of the simulator as a task trainer for improving the skill of durotomy repair is underscored by the improved in-group time comparisons. The magnitude of improvement was influenced by the participants' level of experience. Specifically, PGY-1s experienced the greatest improvement with a reduction in time of 8.34 minutes; PGY-2s showed the next highest improvement at 6.34 minutes; and fellows and attendings were the next at 3.29 minutes. This suggests that the tool is particularly effective for individuals early in their learning curve.

The ability to accurately replicate an in vivo intraoperative scenario with user-controlled intradural pressure enables trainees to confidently refine their skills at varying degrees of difficulty and enhance their technical knowledge. Compared with traditional training methods, this simulator offers a more realistic and immersive experience, potentially leading to improved patient outcomes by allowing trainees to progress through the learning curve of skill acquisition and acquire a greater understanding of technique and decision-making. Currently marketed models can be cost-prohibitive, with high costs for the model or the materials to reset it for continued use. Furthermore, these models are not readily adaptable to anatomical variations in patient spines, as accommodated with imaging-based 3D printing.

### Future Direction and Lessons Learned

With anatomical training models, it is important to maintain correct shapes, landmarks, and anatomical features when rendering out STL files. The model development could have been enhanced if the individual vertebrae were rendered in a series and printed with the patient's natural spine curvature. In addition, future iterations will render the laminectomy with the 3D software before printing to save production time and material waste.

In terms of the fidelity of the model, one issue that was identified by participants was that the needle itself was causing small puncture holes in the Penrose drain, which caused leaks. For future models, adding a coagulant or silicone sealant to the fluid could address this issue. Furthermore, integrating an IV pump to induce pulsatile flow could offer a more realistic simulation, while also allowing for the measurement of the amount of fluid lost. Although the Penrose was selected as a readily accessible, sustainable simulator for dural tissue, biological tissue could benefit more advanced learning. The 25% rate of dural leaks seen in the outcomes of attending surgeons indicates a need for continued evaluation of the fidelity of this simulator when compared with clinical practice.

## CONCLUSION

A simulation model for teaching durotomy repair with 3D modeling of spine segments is an invaluable training tool. The simulator's ability to accurately replicate an in vivo intraoperative scenario enables surgical trainees to confidently refine their technique. Improved technical knowledge, decreased time to repair, and rate of successful repair are some of the potential benefits that come with repeated use of the simulator. By incorporating this simulator into residency and fellowship training programs, orthopedic and neurological surgery trainees can enhance their skills and confidence, potentially leading to improved patient outcomes.

## References

[1] Jastifer JR Gustafson PA. Three-dimensional printing and surgical simulation for preoperative planning of deformity correction in foot and ankle surgery. J Foot Ankle Surg 2017;56(1):191–195. doi:10.1053/j.jfas.2016.01.052 Epub 2016 Mar 5. PMID: 26961413. 26961413

[2] Seymour NE Gallagher AG Roman SA. Virtual reality training improves operating room performance: results of a randomized, double-blinded study. Ann Surg 2002;236(4):458–463; discussion 463-4. doi: 10.1097/00000658-200210000-00008. PMID: 12368674; PMCID: PMC1422600. 12368674 PMC1422600

[3] Aydin A Ahmed K Abe T. SIMULATE Trial Group. Effect of simulation-based training on surgical proficiency and patient outcomes: a randomised controlled clinical and educational trial. Eur Urol 2022;81(4):385–393. Doi: 10.1016/j.eururo.2021.10.030. Epub 2021 Nov 14. Erratum in: Eur Urol. 2022 Dec;82(6):e179. doi: 10.1016/j.eururo.2022.08.036. PMID: 34789393. 34789393 10.1016/j.eururo.2021.10.030

[4] Bernier GV Sanchez JE. Surgical simulation: the value of individualization. Surg Endosc 2016;30(8):3191–3197. doi:10.1007/s00464-016-5021-8. Epub 2016 Jun 23. PMID: 27338581. 27338581

[5] Perone JA Anton NE Gardner AK. Simulation training in surgical education. Curr Surg Rep 2017;5:20. doi:10.1007/s40137-017-0182-5.

[6] Cheng H Clymer JW Po-Han Chen B. Prolonged operative duration is associated with complications: a systematic review and meta-analysis. J Surg Res 2018;229:134–144. doi:10.1016/j.jss.2018.03.022. Epub 2018 Apr 24. PMID: 29936980. 29936980

[7] Meling TR Meling TR. The impact of surgical simulation on patient outcomes: a systematic review and meta-analysis. Neurosurg Rev 2021;44(2):843–854. doi:10.1007/s10143-020-01314-2. Epub 2020 May 13. PMID: 32399730; PMCID: PMC8035110. 32399730 PMC8035110

[8] Ohwaki K Yano E Ishii T. Symptom predictors of cerebrospinal fluid leaks. Can J Neurol Sci 2008;35(4):452–457. doi:10.1017/s0317167100009112 PMID: 18973062. 18973062

[9] Desai A Ball PA Bekelis K. Outcomes after incidental durotomy during first-time lumbar discectomy. J Neurosurg Spine 2011;14(5):647–653. doi:10.3171/2011.1.SPINE10426 Epub 2011 Mar 4. PMID: 21375385; PMCID: PMC4517441. 21375385 PMC4517441

[10] Lin TY Chen WJ Hsieh MK. Postoperative meningitis after spinal surgery: a review of 21 cases from 20,178 patients. BMC Infect Dis 2014;23(14):220. doi:10.1186/1471-2334-14-220.PMID:24755138;PMCID:PMC4013809. PMC4013809 24755138

[11] Wong AP Shih P Smith TR. Comparison of symptomatic cerebral spinal fluid leak between patients undergoing minimally invasive versus open lumbar foraminotomy, discectomy, or laminectomy. World Neurosurg 2014;81(3–4):634–640. doi:10.1016/j.wneu.2013.11.012 Epub 2013 Nov 13. PMID: 24239738. 24239738

[12] Lbukowiec. C2–L5 Anatomically Accurate Spine Model. Thingiverse. https://www.thingiverse.com/thing:7120869. Accessed August 17, 2025.

